# Supplementary material for: Validity and reliability of Indonesian version of the digital screen exposure questionnaire (DSEQ) for young children
Source: PLoS One. 2026 Mar 31;21(3):e0346133. doi: 10.1371/journal.pone.0346133 (PMC13037967; doi:10.1371/journal.pone.0346133)
Supplement: S1 File — (DOCX) [file pone.0346133.s001.docx]

| Department of Community Medicine and School of Public Health,Postgraduate Institute of Medical Education & Research, PGIMER, Chandigarh, 160012website: <http://pgimer.nic.in/> | 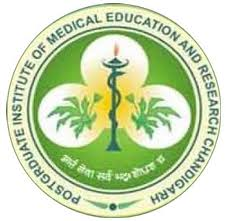 |
| --- | --- |

**BAGIAN I. DETAIL PRIBADI (23 item)**

| 1. Siapa nama anak Anda? ________________________________________ | | | | | | |
| --- | --- | --- | --- | --- | --- | --- |
| 1. Siapakah yang paling sering mengasuh anak?   1) Ibu 2) Ayah 3) Kakek 4) Nenek 5) Lainnya, sebutkan_______________ | | | | | | |
| 1. Apa status pernikahan orangtua?   1) Menikah 2) Janda/Duda 3) Cerai 4) Tinggal terpisah 5) Belum menikah 6) Lainnya, sebutkan__________ | | | | | | |
| 1. Tuliskan tanggal lahir anak: ____/____/________ **ATAU** Perkiraan umur anak (dalam tahun) ________ | | | | | | |
| 1. Jenis kelamin anak:   1) Laki-laki 2) Perempuan | | | | | | |
| 1. Alamat: ____________________________________________________________________ | | | | | | |
| 1. Dimana tempat tinggal anak Anda?   1) Perkotaan 2) Pedesaan 3) Lokasi pengungsian | | | | | | |
| 1. Berapa jumlah ruangan yang ada di rumah: ___________ruangan | | | | | | |
| 1. Apakah Anda memiliki taman atau halaman di dalam atau disekitar rumah untuk anak-anak bermain? | | | | | Iya=1 | Tidak=2 |
| 1. Apa agama Anda?   1) Islam 2) Kristen protestan 3) Katolik 4) Hindu 5) Budhha 6) Konghucu 7) Lainnya, sebutkan_________ | | | | | | |
| 1. Seperti apa jenis keluarga Anda?   1) Keluarga inti 2) Keluarga besar 3) Keluarga dengan 3 generasi (sepupu ibu/ayah) | | | | | | |
|  | **13.1 Hubungan** | **12.2**  **Usia** | **12.3**  ***Pendidikan terakhir** | **12.4**  **^#^Pekerjaan** | | **12.5 “Pendapatan** |
| 12.01 | Ayah |  |  |  | |  |
| 12.02 | Ibu |  |  |  | |  |
| 12.03 | Kepala keluarga selain ayah (jika ada) |  |  |  | |  |
| 12.04 | Kakak/ adik laki-laki/ perempuan |  |  |  | |  |
| 12.05 | Kakak/ adik laki-laki/ perempuan |  |  |  | |  |
| 12.06 | Kakak/ adik laki-laki/ perempuan |  |  |  | |  |
| ***12.3 Pendidikan: 1)** Tidak sekolah: Tidak pernah mengikuti pendidikan formal maupun non-formal. **2)** Sekolah dasar (tidak tamat): Pernah mengikuti pendidikan non-formal atau Sekolah Dasar, tetapi belum menyelesaikannya. **3)** Sekolah menengah pertama (tidak tamat): Telah lulus Sekolah Dasar tetapi belum menyelesaikan Sekolah Menengah Pertama. **4)** Sekolah menengah atas: Telah menyelesaikan Sekolah Menengah Atas hingga lulus. **5)** Diploma: Lulus dari pendidikan Diploma setelah menyelesaikan Sekolah Menengah Atas, termasuk pendidikan teknis atau kejuruan. **6)** Sarjana: Telah menyelesaikan program pendidikan Sarjana (S1). **7)** Profesi atau Lebih Tinggi: Menyelesaikan program pendidikan profesi (seperti dokter, pengacara, dan lainnya) atau pendidikan yang lebih tinggi dari Sarjana (S2/S3).  **#12.4 Pekerjaan: 1)** Anggota dewan, pejabat tinggi perusahaan dan manajer perusahaan **2)** Pekerja profesional **3)** Teknisi dan tenaga ahli madya **4)** Pegawai administrasi **5)** Pekerja terampil dan pekerja toko/pasar/penjualan **6)** Petani dan nelayan **7)** Pengrajin: penenun, tembikar, pelukis, tukang sol sepatu, pembuat sepatu, penjahit, dan lainnya. **8)** Operator dan perakit pabrik dan mesin: industri kecil atau industri rumahan, pekerja industri/pabrik, teknisi listrik, kuli bangunan, tukang ledeng, tukang kayu, pandai emas, pandai besi, montir, dan lainnya. **9)** Pekerja dasar/serabutan: termasuk pekerja di lokasi konstruksi **10)** Tidak bekerja/ibu rumah tangga.  **“12.5** **Pendapatan:** Jumlah penghasilan keluarga perbulan (IDR) **1) >**30.000.000 [12] **2)** 15.000.000-30.000.000 [10]  **3)** 11.000.000-15.000.000 [6] **4)** 7.500.000-11.000.000 [4] **5)** 4.500.000-7.500.000 [3] **6)** 1.500.000-4.500.000 [2] **7)** <1.500.000 [1] | | | | | | |
| 1. Bagaimana status sosial ekonomi keluarga menurut Kuppuswamy (lihat panduan)______________________ | | | | | | |
| 1. Berapa jumlah anggota keluarga yang tinggal dalam satu rumah _______________ | | | | | | |
| 1. Berapa rata-rata pendapatan anggota keluarga (per kapita)_____________________________ | | | | | | |
| 1. Fasilitas perawatan atau penitipan anak. | | | | | | |

| \|  \| 16.02 Berapa hari dalam satu minggu \| 16.03 Durasi/hari (total jam) \| \| --- \| --- \| --- \| \| 16.1 Penitipan anak non-formal (contoh: anggota keluarga, pembantu) \|  \|  \| \| 16.2 Penitipan anak formal (contoh: taman kanak-kanak, kelompok bermain, dan lainya) \|  \|  \| \| 16.3 Dengan orang tua di rumah \|  \|  \| \| 1. Apakah Anda memiliki benda-benda di bawah ini? \| \| \| \|  \| 17.1 Iya=1  atau Tidak=2 \| 17.2 Apakah gadget di letakkan di ruangan tempat anak tidur atau bermain? \| \| - 1. TV dan peralatannya \|  \|  \| \| 17.02 Komputer/Laptop \|  \|  \| \| 17.03 Telepon genggam tanpa jaringan internet \|  \|  \| \| 17.04 Smartphone dengan jaringan internet \|  \|  \| \| 17.05 Perangkat genggam yang dapat digunakan untuk memainkan video game (contoh: tablet) \|  \|  \| \| 17.06 Koneksi internet (kabel internet/ WiFi) \|  \| **Tidak perlu dijawab** \| |
| --- | --- | --- | --- | --- | --- | --- | --- | --- | --- | --- | --- | --- | --- | --- | --- | --- | --- | --- | --- | --- | --- | --- | --- | --- | --- | --- | --- | --- | --- | --- | --- | --- | --- | --- | --- | --- |

| **BAGIAN II. durasi waktu layar dan peralatan digital di rumah (27 ITEM)** |
| --- |

| 1. Jenis aktivitas yang dilakukan anak, durasi dan frekuensinya | 18.1 Menonton TV | 18.2 Bermain dengan smart phone | 18.3 Bermain dengan jenis gawai lainnya, sebutkan | 18.4 Menulis/ menggambar/ mewarnai/ | 18.5 Membaca/ mendengarkan cerita | | | 18.6 Lainnya, sebutkan  ________ |
| --- | --- | --- | --- | --- | --- | --- | --- | --- |
| 18.01 Frekuensi aktivitas anak (poin 18.1-18.6) dalam satu minggu:  1) Tidak Pernah 2) Sangat jarang (Kurang dari sekali) 3) Jarang (1-2 kali)  4) Kadang-kadang (3-4 kali) 5) Sering (5 kali atau lebih) |  |  |  |  |  | | |  |
| 18.02 Berapa rata-rata durasi aktivitas (poin 18.1-18.6) harian anak pada hari kerja/sekolah (menit) |  |  |  |  |  | | |  |
| 18.03 Berapa rata-rata durasi aktivitas (poin 18.1-18.6) harian anak per hari pada hari libur (menit) |  |  |  |  |  | | |  |
| 18.04 Apakah anak-anak didampingi dalam beraktifitas (poin 18.1-18.6) setiap minggu?  1) Tidak Pernah 2) Sangat jarang (Kurang dari sekali) 3) Jarang (1-2 kali)  4) Kadang-kadang (3-4 kali) 5) Sering (5 kali atau lebih) |  |  |  |  |  | | |  |
| 1. Program atau video yang dilihat anak dalam kurun waktu terakhir | A | | B | C | D | | | |
| 1. Berapa lama durasi program atau video tersebut |  | |  |  |  | | | |
| 1. Menurut Anda, apakah penempatan TV di ruangan tem pat anak Anda bermain/tidur dapat meningkatkan atau mengurangi waktu menonton TV bagi anak Anda? Jika tidak, lanjut ke pertanyaan no 23 | | | | | Iya=1 | | Tidak=2 | |
| 1. Jika ya, berapa lama TV tersebut dinyalakan di dalam ruangan tempat anak bermain/tidur (menit)_________ | | | | | | | | |
| 1. Apakah anda memiliki peraturan mengenai kapan, dimana, apa, dan bagaimana untuk bermain gadget? Jika tidak, lanjut ke pertanyaan no 25 | | | | | | Iya=1 | Tidak=2 | |
| 1. Jika iya, seperti apa peraturan yang diterapkan untuk anak-anak dirumah? | | | | | |  |  | |
| 24.1) Hanya program untuk anak-anak yang diperbolehkan | | | | | |  |  | |
| 24.2) Anak dilarang bermain gadget 1 jam sebelum tidur | | | | | |  |  | |
| 24.3) Anak hanya diperbolehkan menonton dengan pengawasan orang dewasa | | | | | |  |  | |
| 24.4) Anak tidak diperbolehkan untuk menonton TV dari jarak dekat | | | | | |  |  | |
| 24.5) Anak diperbolehkan untuk bermain gadget dalam batas waktu tertentu __________________ menit | | | | | |  |  | |
| 24.6) Sebutkan bila ada peraturan lainnya___________________________________________________ | | | | | |  |  | |
| 1. Faktor-faktor terkait media yang berhubungan dengan pengasuh | | | | | | | | |

| \|  \| Ibu \| Ayah \| \| --- \| --- \| --- \| \| 25.01 Rata-rata durasi waktu layar per hari (menit) \|  \|  \| \| 25.02 Bagaimana frekuensi penggunaan gadget dalam 1 minggu  1) Tidak Pernah 2) Sangat jarang (Kurang dari sekali) 3) Jarang (1-2 kali) 4) Kadang-kadang (3-4 kali) 5) Sering (5 kali atau lebih) \|  \|  \| \| 25.03 Gadget yang digunakan  1) TV 2) Komputer 3) Laptop 4) Tablet 6) Smart phone 7) Telepon seluler \|  \|  \| \| 25.04 Rata-rata waktu yang dihabiskan bersama anak dirumah (menit) \|  \|  \| |
| --- | --- | --- | --- | --- | --- | --- | --- | --- | --- | --- | --- | --- | --- | --- | --- |

| BAGIAN III. PERTANYAN MENGENAI AKTIVITAS FISIK (10 ITEM) | | | | | | | |
| --- | --- | --- | --- | --- | --- | --- | --- |
| 1. 01 Durasi rata-rata bermain di luar ruangan per hari pada hari kerja/sekolah (menit) |  | 1. Berapa lama durasi waktu aktivitas di luar ruangan berikut yang dilakukan anak kemarin (menit) | | | | | |
| 26.02 Durasi rata-rata pada hari libur untuk bermain di luar ruangan per hari (menit) |  | - 1. Lari cepat | Ya=1 | Tidak=2 | 27.05 Lompat ringan/kecil | Ya=1 | Tidak=2 |
| 26.03 Total durasi |  | - 1. Berguling |  |  | 27.06 Lompat sedang |  |  |
|  |  | - 1. Menari |  |  | 27.07 Lompat tinggi/jauh |  |  |
|  |  | 27.04 Memanjat |  |  | 27.08 Lainnya________________ |  |  |

**BAGIAN IV. PERILAKU TERKAIT MEDIA (15 ITEM)**

| \| 1. Dalam seminggu, seberapa sering anak memakai gadget untuk beraktifitas di rumah \| Tidak pernah =1 \| Sangat jarang (kurang dari sekali) =2 \| Jarang (1-2 kali) =3 \| Kadang-kadang (3-4 kali) =4 \| Sering (5 kali atau lebih) =5 \| \| --- \| --- \| --- \| --- \| --- \| --- \| \| 1. Anak memakai gadget untuk menyelesaikan tugas pekerjaan rumah/PR \|  \|  \|  \|  \|  \| \| 1. Anak memakai gadget untuk aplikasi video call dengan keluarga atau teman-temannya (skype, whatsapp, dan lainnya) \|  \|  \|  \|  \|  \| \| 1. Anak memakai gadget untuk belajar puisi, pantun, ABC, dan lainnya secara online \|  \|  \|  \|  \|  \| \| 1. Anak memakai gadget untuk belajar berhitung, angka, tabel, secara online \|  \|  \|  \|  \|  \| \| 1. Anak memakai gadget untuk mengenali bentuk, suara, warna yang ditampilkan secara online \|  \|  \|  \|  \|  \| \| 1. Anak memakai gadget untuk belajar tentang ilmu pengetahuan secara online \|  \|  \|  \|  \|  \| \|  \| Tidak pernah =1 \| Sangat jarang (kurang dari sekali) =2 \| Jarang (1-2 kali) =3 \| Kadang-kadang (3-4 kali) =4 \| Sering (5 kali atau lebih) =5 \| \| 1. Anak belajar menggambar dan menulis secara online \|  \|  \|  \|  \|  \| \| 1. Anak bermain video-game \|  \|  \|  \|  \|  \| \| 1. Anak memakai media gadget untuk menonton film (cerita) \|  \|  \|  \|  \|  \| \| 1. Anak menonton program dewasa (sinetron, berita, acara olahraga, film, dan lainnya) \|  \|  \|  \|  \|  \| \| 1. Anak menggunakan untuk mempelajari huruf, kata, kosakata, bahasa secara online \|  \|  \|  \|  \|  \| \| 1. Anak biasanya menonton hal apapun sebagai hiburan (musik, iklan, acara anak-anak, melihat foto, dan lainnya) \|  \|  \|  \|  \|  \| \| 29. Manakah aktivitas dibawah ini yang dilakukan anak ketika menonton TV/? \| \| \| \| \| \| \| 29.1 a Membicarakan tentang program/film \|  \|  \|  \|  \|  \| \| 29.1 b Membicarakan tentang hal lain \|  \|  \|  \|  \|  \| \| 29.1 c Berbicara dengan karakter yang ada di layar \|  \|  \|  \|  \|  \| \| 29.2 Memerankan cerita/memainkan peran sebuah karakter \|  \|  \|  \|  \|  \| \| 29.3 Bernyanyi \|  \|  \|  \|  \|  \| |
| --- | --- | --- | --- | --- | --- | --- | --- | --- | --- | --- | --- | --- | --- | --- | --- | --- | --- | --- | --- | --- | --- | --- | --- | --- | --- | --- | --- | --- | --- | --- | --- | --- | --- | --- | --- | --- | --- | --- | --- | --- | --- | --- | --- | --- | --- | --- | --- | --- | --- | --- | --- | --- | --- | --- | --- | --- | --- | --- | --- | --- | --- | --- | --- | --- | --- | --- | --- | --- | --- | --- | --- | --- | --- | --- | --- | --- | --- | --- | --- | --- | --- | --- | --- | --- | --- | --- | --- | --- | --- | --- | --- | --- | --- | --- | --- | --- | --- | --- | --- | --- | --- | --- | --- | --- | --- | --- | --- | --- | --- | --- | --- | --- | --- | --- | --- | --- | --- | --- | --- | --- |

**BAGIAN V. LITERASI MEDIA ORANG TUA (11 ITEM)**

| 1. Menurut Anda, hal baik apa yang anak-anak pelajari ketika belajar dari layar digital? | | |
| --- | --- | --- |
|  | Iya =1 | Tidak =2 |
| 30.1 Anak mempelajari kebiasaan yang baik |  |  |
| 30.2 Pengetahuan anak meningkat |  |  |
| 30.3 Anak mempelajari keterampilan baru |  |  |
| 30.4 Baik untuk tumbuh kembang anak saya |  |  |
| 30.5 Tidak ada efek positif |  |  |
| 30.6 Lainnya, sebutkan _____________________ |  |  |
| 1. Menurut Anda, apa masalah yang ditimbulkan ketika anak menggunakan layar digital secara berlebihan? | | |
|  | Iya =1 | Tidak =2 |
| 31.1 Anak mulai menirukan apa yang ditontonnya |  |  |
| 31.2 Anak mengalami gangguan tidur |  |  |
| 31.3 Anak mungkin mulai makan makanan yang tidak sehat |  |  |
| 31.4 Anak mungkin menjadi lebih agresif |  |  |
| 31.5 Anak mulai menutup diri dengan sekitar |  |  |
| 31.6 Konsentrasi anak mungkin terganggu |  |  |
| 31.7 Mungkin menyebabkan gangguan perilaku pada anak |  |  |
| 31.8 Mungkin mengganggu penglihatan anak |  |  |
| 31.9 Tidak baik untuk tumbuh kembang anak saya |  |  |
| 31.10 Tidak ada efek negatif |  |  |
| 31.11 Lainnya, sebutkan _________________ |  |  |
