# Supplementary material for: Validity and reliability of Indonesian version of the digital screen exposure questionnaire (DSEQ) for young children
Source: PLoS One. 2026 Mar 31;21(3):e0346133. doi: 10.1371/journal.pone.0346133 (PMC13037967; doi:10.1371/journal.pone.0346133)
Supplement: S1 Checklist — (PDF) [file pone.0346133.s003.pdf]

Digital Screen Exposure Questionnaire (DSEQ)

Petunjuk Pengisian Kuisioner

1. Kuesioner ini berisi pertanyaan-pertanyaan mengenai **paparan layar digital (digital screen exposure)** pada anak.
2. Kuesioner ini **diisi oleh orang tua atau pengasuh** yang memiliki anak berusia **2–5 tahun**.
3. Kuesioner terdiri atas **5 bagian**, dengan estimasi waktu pengisian sekitar **15 menit**.
4. Mohon Bapak/Ibu mengisi setiap pertanyaan **dengan jujur dan sesuai dengan kondisi sebenarnya** yang dialami anak.
5. Tidak ada jawaban benar atau salah. Semua jawaban Bapak/Ibu sangat bermanfaat untuk penelitian ini.

Partisipasi Bapak/Ibu sangat berarti bagi kelancaran dan keberhasilan penelitian ini.

Atas perhatian dan kesediaannya, saya ucapkan terima kasih yang sebesar-besarnya.

Hormat saya,

[Nama Peneliti]

No. Telepon: [Nomor Peneliti]

* Indicates required question

Bagian I. Data Diri

1. 1. Inisial nama anak *

Contoh: mfm

2. 2. Siapakah yang paling sering mengasuh anak? *

Mark only one oval.

- Ibu
- Ayah
- Nenek
- Kakek
- Other: ____________________

3. 3. Status pernikahan orangtua *

Mark only one oval.

- Menikah
- Janda/Duda
- Cerai
- Tinggal terpisah
- Belum menikah
- Other: ____________________

4. 4.1 Tanggal lahir anak *

____

Example: January 7, 2019

5. 4.2 Usia anak *

Contoh: 3 tahun 3 bulan

6. 5. Jenis kelamin anak *

Mark only one oval.

- Laki-laki
- Perempuan

7. 6. Tempat tinggal saat ini *

Contoh : Makassar, Sulawesi Selatan

8. 7. Dimana tempat tinggal anak Anda? *

Mark only one oval.

- Perkotaan
- Pedesaan
- Lokasi pengungsian

9. 8. Jumlah ruangan yang ada di rumah *

Mark only one oval.

- 1-2 ruangan
- 3-4 ruangan
- 5-6 ruangan
- >6 ruangan

10. 9. Apakah Anda memiliki taman atau halaman di dalam atau di sekitar rumah untuk anak-anak bermain? *

Mark only one oval.

- Ya
- Tidak

11. 10. Apa agama Anda? *

Mark only one oval.

- Islam
- Kristen protestan
- Katolik
- Hindu
- Buddha
- Konghucu
- Other: ______

12. 11. Seperti apa jenis keluarga Anda? *

Mark only one oval.

- Keluarga inti (hanya terdiri dari ayah, ibu, dan anak-anak)
- Keluarga besar (ayah, ibu, anak-anak, kakek-nenek/om/tante/sepupu)
- Keluarga dengan 3 generasi (ada sepupu dari pihak ibu/ayah)

13. 12.1 Apakah anak memiliki saudara? *

Mark only one oval per row.

	Tidak ada	1	2	3	≥ 4
**Kakak**	⬭	⬭	⬭	⬭	⬭
**Adik**	⬭	⬭	⬭	⬭	⬭

14. 12.2 Berapa usia saudara anak? *

Mark only one oval per row.

	Tidak ada	<1 tahun	1-3 tahun	4-6 tahun	≥ 7 tahun
**Kakak**	⬭	⬭	⬭	⬭	⬭
**Adik**	⬭	⬭	⬭	⬭	⬭

15. 13.1 Usia ayah dan ibu *

Mark only one oval per row.

	Tidak ada	<20 tahun	20-30 tahun	31-40 tahun	>40 tahun
**Ayah**					
**Ibu**					
**Kepala keluarga selain ayah (jika ada)**					

16. 13.2 Pendidikan terakhir ayah dan ibu *

Mark only one oval per row.

	Tidak ada	Tidak sekolah	SD	SMP	SMA	Diploma	Sarjana	Profesi/Magister (S2)	Doktor (S3)
**Ayah**	⬭	⬭	⬭	⬭	⬭	⬭	⬭	⬭	⬭
**Ibu**	⬭	⬭	⬭	⬭	⬭	⬭	⬭	⬭	⬭
**Kepala keluarga selain ayah (jika ada)**	⬭	⬭	⬭	⬭	⬭	⬭	⬭	⬭	⬭

17. 13.3 Pekerjaan ayah *

Mark only one oval.

- Anggota dewan, pejabat tinggi perusahaan, manajer perusahaan
- Pekerja profesional seperti dokter, dosen, guru, arstitek, pengacara dan lainnya
- Teknisi/tenaga ahli madya
- Pegawai administrasi
- Pedagang/pekerja terampil (orang yang bekerja di toko, pasar, atau bagian penjualan)
- Petani/nelayan
- Pengrajin (orang yang memiliki keterampilan membuat atau memperbaiki barang, seperti penenun, pembuat tembikar, pelukis, tukang sol sepatu, pembuat sepatu, penjahit, dan lainnya)
- Operator/pekerja pabrik (contoh industri kecil/rumahan, pekerja industri/pabrik, teknisi listrik, kuli bangunan, tukang ledeng, tukang kayu, pandai emas, pandai besi, montir, dan lainnya)
- Buruh kasar/buruh harian
- Tidak bekerja/ibu rumah tangga

18. 13.4 Pekerjaan ibu *

Mark only one oval.

- Anggota dewan, pejabat tinggi perusahaan, manajer perusahaan
- Pekerja professional seperti dokter, dosen, guru, dan lainnya
- Teknisi/tenaga ahli madya
- Pegawai administrasi
- Pedagang/pekerja terampil (orang yang bekerja di toko, pasar, atau bagian penjualan)
- Petani dan nelayan
- Pengrajin (orang yang memiliki keterampilan membuat atau memperbaiki barang, seperti penenun, pembuat tembikar, pelukis, tukang sol sepatu, pembuat sepatu, penjahit, dan lainnya)
- Operator/pekerja pabrik (contoh industri kecil/rumahan, pekerja industri/pabrik, teknisi listrik, kuli bangunan, tukang ledeng, tukang kayu, pandai emas, pandai besi, montir, dan lainnya)
- Buruh kasar/buruh harian
- Tidak bekerja/ibu rumah tangga
- Other: ______________________

19. 13.5 Pekerjaan kepala keluarga selain ayah (jika ada)

Mark only one oval.

- Anggota dewan, pejabat tinggi perusahaan, manajer perusahaan
- Pekerja professional seperti dokter, dosen, guru, dan lainnya
- Teknisi/tenaga ahli madya
- Pegawai administrasi
- Pedagang/pekerja terampil (orang yang bekerja di toko, pasar, atau bagian penjualan)
- Petani dan nelayan
- Pengrajin (orang yang memiliki keterampilan membuat atau memperbaiki barang, seperti penenun, pembuat tembikar, pelukis, tukang sol sepatu, pembuat sepatu, penjahit, dan lainnya)
- Operator/pekerja pabrik (contoh industri kecil/rumahan, pekerja industri/pabrik, teknisi listrik, kuli bangunan, tukang ledeng, tukang kayu, pandai emas, pandai besi, montir, dan lainnya)
- Buruh kasar/buruh harian
- Tidak bekerja/ibu rumah tangga
- Other: ______________________

20. 14. Total pendapatan keluarga (ayah, ibu, ataupun kepala keluarga lainnya jika ada) *

Mark only one oval.

- > Rp 30.000.0000
- Rp 15.000.000 - 30.000.000
- Rp 11.000.000 - 15.000.000
- Rp 7.500.000 - 11.000.000
- Rp 4.500.000 - 7.500.000
- Rp 1.500.000 - 4.500.000
- < Rp 1.500.000

21. 15. Jumlah anggota keluarga dalam satu rumah *

Contoh: 3 orang

22. 16.1. Frekuensi fasilitas perawatan atau penitipan anak *

Mark only one oval per row.

	Tidak pernah	1-2 hari/minggu	3-4 hari/minggu	≥ 5 hari/minggu
**Penitipan anak non-formal (contoh anggota keluarga semisal nenek/tante/asisten rumah tangga)**	⬭	⬭	⬭	⬭
**Penitipan anak formal (contoh taman kanak-kanak dan kelompok bermain)**	⬭	⬭	⬭	⬭
**Bersama orang tua**	⬭	⬭	⬭	⬭

23. 16.2. Durasi fasilitas perawatan atau penitipan anak *

Mark only one oval per row.

	Tidak pernah	< 4 jam/hari	4-8 jam/hari	8-12 jam/hari	>12 jam/hari (tidak termasuk waktu tidur malam)
**Penitipan anak non-formal (contoh anggota keluarga semisal nenek/tante/asisten rumah tangga)**	⬭	⬭	⬭	⬭	⬭
**Penitipan anak formal (contoh taman kanak-kanak dan kelompok bermain)**	⬭	⬭	⬭	⬭	⬭
**Bersama orang tua**	⬭	⬭	⬭	⬭	⬭

24. 17.1. Apakah Anda memiliki benda-benda di bawah ini? (Bisa dipilih lebih dari satu) *

Check all that apply.

- [ ] TV dan peralatannya
- [ ] Komputer/laptop
- [ ] Telepon genggam tanpa jaringan internet
- [ ] Smartphone dengan jaringan internet
- [ ] Perangkat genggam yang dapat digunakan untuk memainkan video game (contoh: tablet)
- [ ] Koneksi internet/WiFi

25. 17.2. Manakah benda di bawah ini yang diletakkan di ruangan tempat anak tidur/bermain? (Bisa dipilih lebih dari satu) *

Check all that apply.

- [ ] TV dan peralatannya
- [ ] Komputer/laptop
- [ ] Telepon genggam tanpa jaringan internet
- [ ] Smartphone dengan jaringan internet
- [ ] Perangkat genggam yang dapat digunakan untuk memainkan video game (contoh: tablet)
- [ ] Tidak ada

Bagian II. Durasi Waktu Layar dan Peralatan Digital di Rumah

26. 18.1. Frekuensi aktivitas anak dalam satu minggu *

Mark only one oval per row.

	Tidak pernah	Sangat jarang (<1 kali/minggu)	Jarang (1-2 kali/minggu)	Kadang-kadang (3-4 kali/minggu)	Sering (5 kali atau lebih/minggu)
**Menonton TV**	◯	◯	◯	◯	◯
**Menggunakan smartphone**	◯	◯	◯	◯	◯
**Bermain dengan jenis gawai lainnya seperti laptop/komputer/tablet/playstation, dan semacamnya**	◯	◯	◯	◯	◯
**Menulis/menggambar/mewarnai**	◯	◯	◯	◯	◯
**Membaca/mendengarkan cerita**	◯	◯	◯	◯	◯

27. 18.2. Apakah anak Anda ditemani di sampingnya saat melakukan aktivitas berikut (misal menonton bersama atau menjelaskan tentang apa yang anak tonton)? *

Mark only one oval per row.

	Tidak pernah didampingi	Sangat jarang (<1 kali/minggu)	Jarang (1-2 kali/minggu)	Kadang-kadang (3-4 kali/minggu)	Sering (5 kali atau lebih/minggu)	Tidak melakukan aktivitas tersebut
**Menonton TV**	⬭	⬭	⬭	⬭	⬭	⬭
**Menggunakan smartphone**	⬭	⬭	⬭	⬭	⬭	⬭
**Bermain dengan jenis gawai lainnya seperti laptop/komputer/tablet/playstation, dan semacamnya**	⬭	⬭	⬭	⬭	⬭	⬭
**Menulis/menggambar/mewarnai**	⬭	⬭	⬭	⬭	⬭	⬭
**Membaca/mendengarkan cerita**	⬭	⬭	⬭	⬭	⬭	⬭

28. 18.3. Berapa rata-rata durasi anak/hari pada **hari Senin-Jumat** (hari kerja/sekolah) dalam melakukan aktivitas berikut ini? *

Mark only one oval per row.

	Tidak pernah	<30 menit	30 menit-1 jam/hari	1-2 jam/hari	2-3 jam/hari	3-4 jam/hari	4-5 jam/hari	>5 jam/hari
**Menonton TV**	◯	◯	◯	◯	◯	◯	◯	◯
**Menggunakan smartphone**	◯	◯	◯	◯	◯	◯	◯	◯
**Bermain dengan jenis gawai lainnya seperti laptop/komputer/tablet/playstation, dan semacamnya**	◯	◯	◯	◯	◯	◯	◯	◯
**Menulis/menggambar/mewarnai**	◯	◯	◯	◯	◯	◯	◯	◯
**Membaca/mendengarkan cerita**	◯	◯	◯	◯	◯	◯	◯	◯

29. 18.4. Berapa rata-rata durasi anak/hari pada **hari Sabtu dan Minggu** (hari libur) dalam melakukan aktivitas berikut ini? *

Mark only one oval per row.

	Tidak pernah	<30 menit	30 menit-1 jam/hari	1-2 jam/hari	2-3 jam/hari	3-4 jam/hari	4-5 jam/hari	>5 jam/hari
**Menonton TV**	⬭	⬭	⬭	⬭	⬭	⬭	⬭	⬭
**Menggunakan smartphone**	⬭	⬭	⬭	⬭	⬭	⬭	⬭	⬭
**Bermain dengan jenis gawai lainnya seperti laptop/komputer/tablet/playstation, dan semacamnya**	⬭	⬭	⬭	⬭	⬭	⬭	⬭	⬭
**Menulis/menggambar/mewarnai**	⬭	⬭	⬭	⬭	⬭	⬭	⬭	⬭
**Membaca/mendengarkan cerita**	⬭	⬭	⬭	⬭	⬭	⬭	⬭	⬭

30. 19. Jenis program atau video apa yang biasa anak nonton (bisa memilih lebih dari satu) *

Check all that apply.

- [ ] Youtube
- [ ] Youtube Kids
- [ ] Program atau video di TV
- [ ] Video pendek dari media sosial (TikTok/Instagram Reels/Youtube Shorts)
- [ ] Hanya program atau video tertentu yang telah disortir oleh orang tua
- [ ] Other: ____________________

31. 20. Berapa lama rata-rata durasi program/video tersebut?

Check all that apply.

	<1 menit	1-5 menit	5-10 menit	10-15 menit	15-30 menit	>30 menit
**Youtube**	☐	☐	☐	☐	☐	☐
**Youtube Kids**	☐	☐	☐	☐	☐	☐
**Program atau video di TV**	☐	☐	☐	☐	☐	☐
**Video pendek dari media sosial (TikTok/Instagram Reels/Youtube Shorts)**	☐	☐	☐	☐	☐	☐
**Hanya program atau video tertentu yang telah disortir oleh orang tua**	☐	☐	☐	☐	☐	☐

32. 21. Menurut Anda, apakah penempatan TV di ruangan tempat anak Anda bermain/tidur dapat meningkatkan atau mengurangi waktu menonton TV bagi anak Anda? *(Jika tidak, lanjut ke pertanyaan no. 23)* *

Mark only one oval.

- Ya
- Tidak

33. 22. Jika jawaban Anda pada **No.21** Ya, berapa lama TV tersebut dinyalakan di dalam ruangan tempat anak bermain/tidur (menit)

____

34. 23. Apakah Anda memiliki peraturan mengenai kapan, dimana, apa, dan bagaimana tentang bermain gadget? *(Jika tidak, lanjut ke pertanyaan no. 25)* *

Mark only one oval.

- Ya
- Tidak

35. 24. Jika jawaban Anda pada **No.23 Ya**, centang peraturan yang Anda terapkan (bisa memilih lebih dari satu)

Check all that apply.

- [ ] Hanya program untuk anak-anak yang diperbolehkan
- [ ] Anak dilarang bermain gadget 1 jam sebelum tidur
- [ ] Anak hanya diperbolehkan menonton dengan orang dewasa disampingnya
- [ ] Anak tidak diperbolehkan untuk menonton TV dari jarak dekat
- [ ] Anak hanya diperbolehkan bermain gadget kurang dari 1 jam
- [ ] Anak hanya diperbolehkan bermain gadget kurang dari 2 jam
- [ ] Other: ____________________

36. 25.1. Rata-rata durasi waktu layar Ayah dan Ibu perhari *

Mark only one oval per row.

	Tidak pernah	<1 jam	1-2 jam	2-3 jam	3-4 jam	4-5 jam	>5 jam
**Ayah**	◯	◯	◯	◯	◯	◯	◯
**Ibu**	◯	◯	◯	◯	◯	◯	◯

37. 25.2. Frekuensi penggunaan gadget Ayah dan Ibu dalam satu minggu *

Mark only one oval per row.

	Tidak pernah	Sangat jarang (<1 kali/minggu)	Jarang (1-2 kali/minggu)	Kadang-kadang (3-4 kali/minggu)	Sering (5 kali atau lebih)
**Ayah**					
**Ibu**					

38. 25.3. Gadget yang digunakan Ayah dan Ibu (bisa pilih lebih dari satu) *

Check all that apply.

	TV	Komputer	Laptop	Tablet	Smartphone	Telepon seluler
**Ayah**						
**Ibu**						

39. 25.4. Rata-rata durasi waktu Ayah dan Ibu bermain bersama anak di rumah/hari (tanpa sambil menonton TV, bekerja, atau menggunakan HP)? *

Mark only one oval per row.

	Tidak pernah	<30 menit	30-60 menit	1-2 jam	2-3 jam	>3 jam
**Ayah**	⬭	⬭	⬭	⬭	⬭	⬭
**Ibu**	⬭	⬭	⬭	⬭	⬭	⬭

Bagian III. Pertanyaan Mengenai Aktivitas Fisik

40. 26. Durasi rata-rata bermain di luar ruangan perhari pada hari kerja dan hari libur *

Mark only one oval per row.

	Tidak bermain di luar ruangan	<1 jam	1-2 jam	2-3 jam	>3 jam
**Hari kerja (Senin-Jumat)**	⬭	⬭	⬭	⬭	⬭
**Hari libur (Sabtu dan Minggu)**	⬭	⬭	⬭	⬭	⬭

41. 27. Berapa lama durasi waktu aktivitas di luar ruangan berikut yang dilakukan anak **kemarin** *

Mark only one oval per row.

	Tidak melakukan	< 10 menit	10-30 menit	> 30 menit
**Lari cepat**	◯	◯	◯	◯
**Berguling**	◯	◯	◯	◯
**Menari**	◯	◯	◯	◯
**Memanjat**	◯	◯	◯	◯
**Lompat ringan/kecil**	◯	◯	◯	◯
**Lompat sedang**	◯	◯	◯	◯
**Lompat tinggi**	◯	◯	◯	◯

Bagian IV. Perilaku Terkait Media

Petunjuk Pengisian.

Berikut ini terdapat beberapa pernyataan terkait kebiasaan anak Anda.

Mohon pilih angka dari 1 hingga 5 yang paling sesuai dengan kondisi anak Anda dalam **satu minggu terakhir**, dengan panduan sebagai berikut:

1 = Tidak pernah

2 = Sangat jarang (kurang dari sekali dalam seminggu)

3 = Jarang (1–2 kali dalam seminggu)

4 = Kadang-kadang (3–4 kali dalam seminggu)

5 = Sering (5 kali atau lebih dalam seminggu)

42. 28.1. Anak memakai gadget untuk menyelesaikan tugas pekerjaan rumah/PR *

Mark only one oval.

	1	2	3	4	5	
Tida	◯	◯	◯	◯	◯	Sering

43. 28.2. Anak memakai gadget untuk aplikasi video call dengan keluarga atau teman-temannya (skype, whatsapp, dan lainnya) *

Mark only one oval.

	1	2	3	4	5	
Tida	◯	◯	◯	◯	◯	Sering

44. 28.3. Anak memakai gadget untuk belajar puisi, pantun, ABC, dan lainnya secara online *

Mark only one oval.

	1	2	3	4	5	
Tida	◯	◯	◯	◯	◯	Sering

45. 28.4. Anak memakai gadget untuk belajar berhitung, angka, tabel, secara online *

Mark only one oval.

	1	2	3	4	5	
Tida	◯	◯	◯	◯	◯	Sering

46. 28.5. Anak memakai gadget untuk mengenali bentuk, suara, warna, yang ditampilkan secara online *

Mark only one oval.

	1	2	3	4	5	
Tida	◯	◯	◯	◯	◯	Sering

47. 28.6. Anak memakai gadget untuk belajar tentang ilmu pengetahuan secara online *

Mark only one oval.

	1	2	3	4	5	
Tida	◯	◯	◯	◯	◯	Sering

48. 28.7. Anak belajar menggambar dan menulis secara online *

Mark only one oval.

	1	2	3	4	5	
Tida	◯	◯	◯	◯	◯	Sering

49. 28.8. Anak bermain video-game *

Mark only one oval.

	1	2	3	4	5	
Tida	◯	◯	◯	◯	◯	Sering

50. 28.9. Anak memakai media gadget untuk menonton film (cerita) *

Mark only one oval.

	1	2	3	4	5	
Tida	◯	◯	◯	◯	◯	Sering

51. 28.10. Anak menonton program dewasa (sinetron, berita, acara olahraga, film, dan lainnya) *

Mark only one oval.

	1	2	3	4	5	
Tida	◯	◯	◯	◯	◯	Sering

52. 28.11. Anak menggunakan untuk mempelajari huruf, kata, kosakata, bahasa secara online *

Mark only one oval.

	1	2	3	4	5	
Tida	◯	◯	◯	◯	◯	Sering

53. 28.12. Anak biasanya menonton hal apapun sebagai hiburan (musik, iklan, acara anak-anak, melihat foto, dan lainnya) *

Mark only one oval.

	1	2	3	4	5	
Tida	◯	◯	◯	◯	◯	Sering

54. 29.1. Apakah anak Anda membicarakan tentang program/film ketika menonton TV/gadget? *

Mark only one oval.

	1	2	3	4	5	
Tida	◯	◯	◯	◯	◯	Sering

55. 29.2. Apakah anak Anda membicarakan tentang hal lain ketika menonton TV/gadget? *

Mark only one oval.

	1	2	3	4	5	
Tida	◯	◯	◯	◯	◯	Sering

56. 29.3. Apakah anak Anda berbicara dengan karakter yang ada di layar ketika menonton TV/gadget? *

Mark only one oval.

	1	2	3	4	5	
Tida	◯	◯	◯	◯	◯	Sering

57. 29.4. Apakah anak Anda memerankan cerita/memainkan peran sebuah karakter ketika menonton TV/gadget? *

Mark only one oval.

	1	2	3	4	5	
Tida	◯	◯	◯	◯	◯	Sering

58. 29.5. Apakah anak Anda bernyayi ketika menonton TV/gadget? *

Mark only one oval.

	1	2	3	4	5	
Tida	◯	◯	◯	◯	◯	Sering

Bagian V. Literasi Media Orang Tua

59. 30. Menurut Anda, hal baik apa yang anak-anak pelajari ketika belajar dari layar digital? (Bisa pilih lebih dari satu) *

Check all that apply.

- [ ] Anak mempelajari kebiasaan yang baik
- [ ] Pengetahuan anak meningkat
- [ ] Anak mempelajari keterampilan baru
- [ ] Baik untuk tumbuh kembang anak saya
- [ ] Tidak ada efek positif
- [ ] Other: ____________________

60. 31. Menurut Anda, apa masalah yang ditimbulkan ketika anak menggunakan layar digital secara berlebihan? (Bisa pilih lebih dari satu) *

Check all that apply.

- [ ] Anak mulai menirukan apa yang ditontonnya
- [ ] Anak mengalami gangguan tidur
- [ ] Anak mungkin mulai makan makanan yang tidak sehat
- [ ] Anak mungkin menjadi lebih agresif
- [ ] Anak mulai menutup diri dengan sekitar
- [ ] Konsentrasi anak mungkin terganggu
- [ ] Mungkin menyebabkan gangguan perilaku pada anak
- [ ] Mungkin menganggu penglihatan anak
- [ ] Tidak baik untuk tumbuh kembang anak saya
- [ ] Tidak ada efek negatif
- [ ] Other: ______________________

This content is neither created nor endorsed by Google.

Google Forms
