# Supplementary material for: Validity and reliability of Indonesian version of the digital screen exposure questionnaire (DSEQ) for young children
Source: PLoS One. 2026 Mar 31;21(3):e0346133. doi: 10.1371/journal.pone.0346133 (PMC13037967; doi:10.1371/journal.pone.0346133)
Supplement: Attachment — Submitted filename: Response to Reviewers.docx [file pone.0346133.s005.docx]

**Response to Reviewers**

Thank you for your insightful comments and valuable suggestions, which have significantly improved our manuscript. We have addressed all comments below and explained the revisions made.

*The explanation (page, number, and line) was based on Revised Manuscript with Track Changes file.*

A. **From Editor**
1. Please ensure that your manuscript meets PLOS ONE's style requirements, including those for file naming. The PLOS ONE style templates can be found at <https://journals.plos.org/plosone/s/file?id=wjVg/PLOSOne_formatting_sample_main_body.pdf> and <https://journals.plos.org/plosone/s/file?id=ba62/PLOSOne_formatting_sample_title_authors_affiliations.pdf>

**Response:** We have carefully checked the manuscript and ensured that it meets all PLOS ONE style and formatting requirements.

2. Please include a complete copy of PLOS’ questionnaire on inclusivity in global research in your revised manuscript. Our policy for research in this area aims to improve transparency in the reporting of research performed outside of researchers’ own country or community. The policy applies to researchers who have travelled to a different country to conduct research, research with Indigenous populations or their lands, and research on cultural artefacts. The questionnaire can also be requested at the journal’s discretion for any other submissions, even if these conditions are not met.  Please find more information on the policy and a link to download a blank copy of the questionnaire here: <https://journals.plos.org/plosone/s/best-practices-in-research-reporting>. Please upload a completed version of your questionnaire as Supporting Information when you resubmit your manuscript.

**Response:** We have completed the PLOS questionnaire on inclusivity in global research and included it as Supporting Information (S1 Checklist). In addition, a related subsection has been added to the Methods section (page 10, lines 221–224).

“Additional information regarding the ethical, cultural, and scientific considerations specific to inclusivity in global research is included in the Supporting Information (S1 Checklist).”

3. Please include your full ethics statement in the ‘Methods’ section of your manuscript file. In your statement, please include the full name of the IRB or ethics committee who approved or waived your study, as well as whether or not you obtained informed written or verbal consent. If consent was waived for your study, please include this information in your statement as well.

**Response:** We have included the complete ethics statement in the Methods section (page 10, lines 215–220).

“This study was approved by the Ethics Committee of Tokyo Metropolitan University, Arakawa Campus (Approval No. 24086). All participants gave their consent after being informed about the aims and procedures of the study. In addition, permission was obtained from the original author or developer of the Digital Screen Exposure Questionnaire (DSEQ) to translate and culturally adapt the instrument for use in the Indonesian context.”

4. When completing the data availability statement of the submission form, you indicated that you would make your data available on acceptance. We strongly recommend all authors decide on a data sharing plan before acceptance, as the process can be lengthy and hold up publication timelines. Please note that, though access restrictions are acceptable now, your entire data will need to be made freely accessible if your manuscript is accepted for publication. This policy applies to all data except where public deposits would breach compliance with the protocol approved by your research ethics board. If you are unable to adhere to our open data policy, please kindly revise your statement to explain your reasoning and we will seek the editor's input on an exemption. Please be assured that once you have provided your new statement, the assessment of your exemption will not hold up the peer review process.

**Response:** We have revised the data availability statement to clarify our data-sharing plan in accordance with PLOS ONE’s open data policy.

5. Please amend your authorship list in your manuscript file to include author [Jusriani Jusriani](mailto:jusriani20.ani@gmail.com).

**Response:** The author’s name has been corrected and updated to “Jusriani Jusriani” in the manuscript file (page 1 line 3).

6. Please amend the manuscript submission data (via Edit Submission) to include author Jusriani.

**Response:** The manuscript submission data have been updated to include Jusriani Jusriani as an author.

7. If the reviewer comments include a recommendation to cite specific previously published works, please review and evaluate these publications to determine whether they are relevant and should be cited. There is no requirement to cite these works unless the editor has indicated otherwise.

**Response:** We have reviewed and cited the article recommended by Reviewer 1 (Yetiş et al., 2025) in the revised manuscript.

B. From Reviewer 1
1. The abstract can briefly summarize which items in the translated scale have been changed. The test-retest sample size (n=31) must be clearly stated in the abstract (the authors have mentioned it, but it could be made more visible).

**Response:** We have clearly stated the test–retest sample size (n = 31) in the abstract (page 2, line 33).

“Reliability was assessed based on the COSMIN guidelines and previous studies, with internal consistency measured using Cronbach’s alpha and test–retest reliability assessed in a subsample of 31 caregivers using the intraclass correlation coefficient (ICC).”

2. The main message could be more impactful: “This study is the first cultural adaptation of the DSEQ in Indonesia.”

**Response:** We have added the statement highlighting that this study is the first cultural adaptation of the DSEQ in Indonesia to the abstract (page 2, line 34).

“This study is the first cultural adaptation of the DSEQ in Indonesia and demonstrates good face and content validity, as confirmed by expert evaluations.”

3. More could be said about the effects of parental guidance, content type, and media literacy on development.

**Response:** Additional discussion on parental guidance, media content, and media literacy has been added to the Introduction (page 4, lines 76–84).

“The most common activity performed by children aged <5 years on mobile devices was watching videos (70.8%). Additionally, 59.6% of the parents allowed their children to use mobile devices while engaging in daily tasks and domestic chores. Furthermore, 91.5% of parents reported that they had not been informed by pediatricians regarding the potential negative effects of mobile device use on their children’s development [2]. When children use mobile devices while their parents are occupied with daily tasks and household chores, insufficient parental supervision can lead to inappropriate content exposure and excessive screen usage. Therefore, pediatricians should inquire regarding the duration of children’s screen time, provide appropriate advice, and warn parents of the risk of excessive media use [2,18].”

4. The research hypothesis is not clearly stated at the end of the introduction. Suggestion: “It is hypothesized that the Indonesian DSEQ will demonstrate good validity and reliability.”

**Response:** The research hypothesis has been clearly stated at the end of the Introduction (page 5, lines 113–114).

“We hypothesized that the Indonesian DSEQ would have good validity and reliability.”

5. A minimum sample size calculation has been made in accordance with the COSMIN guidelines. However, it would be more accurate to include 3-5 or 10 people in each question in the survey. You should write the power analysis accordingly. You should also reference the following article. References 1: Yetiş, A., Canli, M., Yildiz, N. T., Kocaman, H., Yildirim, H., Kuzu, Ş., ... & Duran, S. (2025). Investigation of the validity, reliability and psychometric properties of the Turkish version of the Ottawa sitting scale in patients with Parkinson's disease. Scientific Reports, 15(1), 2628.

**Response:** We have added a new subsection describing the sample size rationale based on COSMIN guidelines and item-to-participant ratios, and cited the recommended article (Yetiş et al., 2025) in the Methods section (page 6–7, lines 131–139).

“However, a more accurate approach would be to include three to five or up to 10 participants per questionnaire item [25]. A previous study assessed internal consistency using 52 items across domains 2–4 [20]. One item from domain 3 was removed from the online questionnaire (Table 2). Therefore, an internal consistency analysis was conducted using 51 items. Accordingly, the minimum required sample size was 153.”

6. Convenience + snowball sampling limits representativeness. This limitation should be more strongly addressed in the discussion.

**Response:** We have expanded the discussion of sampling limitations and explained the rationale for using convenience and snowball sampling in the Discussion section (page 25–26, lines 373–380).

“Second, the data were collected using convenience and snowball sampling, which may have introduced sampling bias. Third, the screen time data were based on parental reports (questionnaire-based) and may have been subject to recall or reporting bias. However, convenience and snowball sampling and questionnaire-based measures are widely used and considered acceptable in similar contexts, and have been applied in several previous studies on screen time [39,40,41,42]. Moreover, convenience and snowball sampling were employed to facilitate participant access, increase the sample size, and enhance participant trust, particularly because the survey was administered online and link-based scams have become common in Indonesia.”

7. The rationale for inclusion criteria should be explained (e.g., why only 2–5 years?)

**Response:** The rationale for selecting children aged 2–5 years has been clarified in the Participants subsection of the Methods section (page 6, lines 124–128).

“This age range was selected because the developers of the DSEQ designed the instrument specifically for children aged 2–5 years [20]. In addition, previous studies have reported that the association between excessive screen time and developmental or behavioral problems is stronger in preschool-aged children than in older children or adolescents [14], and that excessive screen time generally increases with age [7,8].”

8. Variables such as parental education level and socioeconomic status could be reported more systematically.

**Response:** The parental education level has been reported in greater detail, and additional rows describing socioeconomic status have been added to the Results section (pages 12 and 13).

| 10 | Mother education |  |
| --- | --- | --- |
|  | Primary school certificate | 2 (1.2%) |
|  | Middle school certificate | 2 (1.2%) |
|  | High school certificate | 46 (26.9%) |
|  | Intermediate or diploma | 18 (10.5%) |
|  | Graduate | 71 (41.5%) |
|  | Professional degree or with honors | 32 (18.7%) |
| 11 | Father education |  |
|  | Primary school certificate | 3 (1.8%) |
|  | Middle school certificate | 7 (4.1%) |
|  | High school certificate | 55 (32.1%) |
|  | Intermediate or diploma | 5 (2.9%) |
|  | Graduate | 67 (39.2%) |
|  | Professional degree or with honors | 34 (19.9%) |
| 12 | Socioeconomic status |  |
|  | Upper middle (II) | 100 (58.5%) |
|  | Lower middle (III) | 52 (30.4%) |
|  | Upper lower (IV) | 19 (11.1%) |

9. The “acceptable to excellent” statements for Cronbach's alpha should be defined by classification.

**Response:** A new Reliability subsection has been added to the Methods section, including the classification criteria for Cronbach’s alpha values (page 9, lines 198–200).

“Furthermore, the Cronbach’s alpha values were interpreted using commonly applied thresholds: excellent (>0.90), good (>0.80), acceptable (>0.70), questionable (>0.60), poor (>0.50), and unacceptable (<0.50) [29].”

10. The explanation for Domain 3 is confusing; it could be more clearly stated which items were excluded.

**Response:** We have clarified which item was excluded from Domain 3 in the Results section (page 18, line 269-270). The exclusion criterion was total duration of physical activity.

“In addition, because two items in Domain 1 (sociodemographic characteristics) and one item (total duration) in Domain 3 (physical activity) were removed from the online questionnaire (Table 2), the total number of items for the online version was 83, whereas the paper-based version used for interview method surveys retained all 86 items.”

11. ICC reporting should be more standardized: the ICC model (ICC 2.1 or 3.1?) should be specified.

**Response:** The ICC model used (ICC model specification) has been clearly reported in the Statistical Analysis subsection of the Methods section (page 9, lines 202 and 203).

The test–retest reliability was assessed using the intraclass correlation coefficient (ICC) based on a two-way mixed-effects model with consistency for single measurements (ICC 3.1).”

12. The confidence interval (CI 95%) should be provided.

**Response:** A new column with 95% confidence intervals has been added to the Results (pages 20–21).

| **Domain and Item** | | | **ICC** | | **95% CI** | | **Sig.** |
| --- | --- | --- | --- | --- | --- | --- | --- |
| **Domain 2: Screen exposure and home media environment** | | | | | | | |
| 1 | What is the frequency of watching television in a typical week? | 0.663 | | 0.409 – 0.822 | | <0.001 | |
| 2 | Duration of watching television on a typical working day? | 0.745 | | 0.535 – 0.868 | | <0.001 | |
| 3 | Duration of watching television on a typical holiday? | 0.874 | | 0.755 – 0.937 | | <0.001 | |
| 4 | Does the child watch television supervision frequently by an adult? | 0.514 | | 0.201 – 0.732 | | 0.001 | |
| 5 | What is the frequency of using smartphone in a typical week? | 0.618 | | 0.342 – 0.795 | | <0.001 | |
| 6 | Duration of using smartphone on a typical working day? | 0.656 | | 0.398 – 0.818 | | <0.001 | |
| 7 | Duration of using smartphone on a typical holiday? | 0.521 | | 0.210 – 0.737 | | 0.001 | |
| 8 | Does the child use smartphone supervision frequently by an adult? | 0.539 | | 0.234 – 0.748 | | <0.001 | |
| 9 | What is the frequency of using other gadgets in a typical week? | 0.541 | | 0.237 – 0.749 | | <0.001 | |
| 10 | Duration of other gadgets on a typical working day? | 0.561 | | 0.264 – 0.761 | | <0.001 | |
| 11 | Duration of other gadgets on a typical holiday? | 0.647 | | 0.385 – 0.813 | | <0.001 | |
| 12 | Does the child use other gadgets supervision frequently by an adult? | 0.601 | | 0.319 – 0.786 | | <0.001 | |
| 13 | Do you have any rules regarding when, where, what & how to watch digital screen? | 0.614 | | 0.337 – 0.793 | | <0.001 | |
| 14 | Average duration of screen time per day of the mother | 0.674 | | 0.424 – 0.828 | | <0.001 | |
| 15 | Average duration of screen time per day of the father | 0.802 | | 0.629 – 0.899 | | <0.001 | |
| **Domain 3: Level of physical activity** | | | | | | | |
| 16 | Average duration of outside play per day on working/school days | 0.679 | | 0.433 – 0.831 | | <0.001 | |
| 17 | Average duration of outside play per day on holidays | 0.705 | | 0.471 – 0.846 | | <0.001 | |
| **Domain 4: Media behaviors of the child** | | | | | | | |
| 18 | The child uses for completing their homework assignments | 0.946 | | 0.891 – 0.973 | | <0.001 | |
| 19 | The child uses video calling applications to talk to family or friends | 0.796 | | 0.619 – 0.896 | | <0.001 | |
| 20 | The child uses poems rhymes ABC etc. online | 0.549 | | 0.247 – 0.754 | | <0.001 | |
| 21 | The child uses math numbers of tables online | 0.526 | | 0.535 – 0.868 | | 0.001 | |
| 22 | The child uses to recognize shapes sounds colors when shown online | 0.535 | | 0.228 – 0.745 | | <0.001 | |
| 23 | The child learns various sciences online | 0.780 | | 0.592 – 0.887 | | <0.001 | |
| 24 | The child learns to draw write online | 0.656 | | 0.397 – 0.818 | | <0.001 | |
| 25 | The child plays video games | 0.546 | | 0.242 – 0.752 | | <0.001 | |
| 26 | The child uses digital media gadgets to watch stories | 0.678 | | 0.430 – 0.830 | | <0.001 | |
| 27 | The child to watch adult programs | 0.905 | | 0.813 – 0.953 | | <0.001 | |
| 28 | The child uses to learns letters words vocabulary language online | 0.59 | | 0.303 – 0.779 | | <0.001 | |
| 29 | The child uses to watch random things for enjoyment | 0.584 | | 0.294 – 0.775 | | <0.001 | |

13. The normality of the data or the validation of the test-retest interval should be explained.

**Response:** We have explained the rationale for the test–retest interval in the Methods section (page 9, lines 192–198). Previous studies have used 10-day and 14-day intervals. In our study, participants were instructed to complete the retest after 10 days; however, some participants completed it after 11–12 days, which we considered acceptable. This interval was selected to reduce recall bias while minimizing the likelihood of substantial changes in children’s daily routines.

“The original questionnaire showed test–retest reliability in 30 participants over a 10-day interval [20]. Similarly, another study that developed a questionnaire to assess leisure-time screen-based media use in children aged 6–10 years has reported test–retest reliability in 35 participants over a 2-week interval [28]. In this study, test–retest reliability was assessed with 31 participants over an interval of 10–12 days, which met the minimum sample size requirement, and the interval was aligned with previous studies [20,27,28].”

14. The discussion could be more structured: Main findings, Literature comparison, Methodological advantages, Limitations, Conclusion and recommendations

**Response:** The Discussion has been restructured to clearly address the main findings, comparisons with previous studies, methodological advantages, limitations, conclusions, and recommendations.

“Comparisons with other countries were limited because validity and reliability evidence is currently available only from the original developer’s study.” (page 23, lines 306 and 307).

The methodological advantages are discussed on page 24 (lines 340–348).

“In addition to questionnaires, screen exposure in children can be assessed such as through diaries, electronically prompted sampling, direct observation, recording devices (fixed or portable cameras and audio recorders), screen devices for onboard logging, and remote digital trace logging. Each method has its advantages and disadvantages. However, methods with higher accuracy and lower bias often incur higher costs, and may raise privacy concerns for children and their families, making it difficult to recruit participants [32]. Therefore, using a questionnaire is considered appropriate because it imposes a low burden on researchers and participants. However, this still depends on the ability of the researcher and the willingness of the participants, as methods with lower bias are preferable whenever feasible.”

The conclusions and future research directions are presented on page 26 (lines 382–392).

“The Indonesian version of the DSEQ showed good face and content validity as confirmed by experts, acceptable to excellent internal consistency across domains, and moderate-to-high test–retest reliability. Thus, the Indonesian DSEQ (online version) may be a valid and reliable tool for assessing digital screen exposure in early childhood and may be useful for research, clinical practice, and public health programs in Indonesia. Future studies are recommended to evaluate the Indonesian DSEQ in larger and more diverse populations across Indonesia to enhance its generalizability and to evaluate the validity and reliability of paper-based questionnaires using the interview method. Moreover, the Indonesian DSEQ could be used in future research to investigate the association between digital screen exposure and various aspects of child development.”

15. There are too many operational details; the discussion should focus on scientific interpretations.

**Response:** We have removed excessive operational details from the Discussion section (pages 23 and 24, lines 324–334) and moved them to the Methods section, specifically to the Reliability subsection (page 9, lines 189–200 multidisciplinary panel of professionals specializing in child development, including pediatrics, child psychology, physical therapy, occupational therapy, speech therapy, and early childhood education).

The following sentences have been moved and revised from the Discussion to the Methods: “The minimum required number of participants for test-retest reliability is 30, and ICC values < 0.5, 0.6–0.75, 0.76–0.9, and > 0.9 indicate poor, moderate, good, and excellent reliabilities, respectively [27]. The original questionnaire showed test–retest reliability in 30 participants over a 10-day interval [20]. Similarly, another study that developed a questionnaire to assess leisure-time screen-based media use in children aged 6–10 years has reported test–retest reliability in 35 participants over a 2-week interval [28]. In this study, test–retest reliability was assessed with 31 participants over an interval of 10–12 days, which met the minimum sample size requirement, and the interval was aligned with previous studies [20,27,28]. Furthermore, the Cronbach’s alpha values were interpreted using commonly applied thresholds: excellent (>0.90), good (>0.80), acceptable (>0.70), questionable (>0.60), poor (>0.50), and unacceptable (<0.50) [29]”

16. The relationship between socioeconomic status and parental screen-time could be addressed.

**Response:** A new subsection on socioeconomic status and parental screen time has been added to the Results section (page 22, lines 292–298).

**“**In the chi-square analysis, socioeconomic status was significantly associated with fathers’ screen time (χ² = 10.1, p = 0.006), indicating that screen time patterns differed across socioeconomic groups. By contrast, no significant association was observed between socioeconomic status and mothers’ screen time (χ² = 1.78, p = 0.41).”

| No | Characteristics | n (%) | | | | P value |
| --- | --- | --- | --- | --- | --- | --- |
|  |  | All participants | Socioeconomic status | | |  |
|  |  |  | II | III | IV |  |
|  |  | n=171 | n=100 | n=52 | n=19 |  |
| 1 | Mother screen time |  |  |  |  | 0.41 |
|  | <3 h | 116 (67.8%) | 65 (65%) | 39 (75%) | 12 (63.2%) |  |
|  | >3 h | 55 (32.2%) | 35 (35%) | 13 (25%) | 7 (36.8%) |  |
| 2 | Father screen time |  |  |  |  | 0.006* |
|  | <3 h | 94 (55%) | 46 (46%) | 38 (73.1%) | 10 (52.6%) |  |
|  | >3 h | 77 (45%) | 54 (54%) | 14 (26.9%) | 9 (47.4%) |  |

Statistical significance was set at p < 0.05.

Moreover, corresponding interpretations have been included in the Discussion (page 24–25, lines 349–362).

“Another finding of this study was that socioeconomic status was significantly associated with fathers’ screen time but not with mothers’ screen time. Fathers with higher socioeconomic status (level II) were more likely to report screen times exceeding 3 h, suggesting that paternal screen use may vary according to socioeconomic conditions. By contrast, mothers’ screen time showed relatively similar patterns across socioeconomic levels, which may be related to their comparable caregiving roles and daily routines. Although direct comparisons of fathers’ and mothers’ screen time by socioeconomic status remain limited, the findings vary across countries. For example, a previous European study has reported that parents from families with a lower socioeconomic status applied more consistent rules regarding gaming and watched more television near their children; these parenting practices were associated with longer screen time [36]. In addition, studies from China have indicated that children and adolescents from lower socioeconomic backgrounds are more likely to have higher levels of screen time [37], highlighting the role of the socioeconomic context in shaping screen-related behaviors within families.”

C. **From Reviewer 2**
1. Abstract

The abstract is generally clear and acceptable. Minor refinement is suggested regarding the reporting of test–retest reliability. It would be helpful to clarify whether the reported ICC values apply consistently across all questionnaire domains and to briefly contextualize domains with lower ICC values.

**Response:** We have clarified that the reported ICC values apply to specific questionnaire domains and provided contextual explanations for domains with lower ICC values in the abstract (page 2, lines 38–40).

“Test–retest reliability across the three domains was moderate to high (ICC values: 0.514–0.946), with lower ICC values observed for the item related to parental supervision while watching television, which may vary owing to differences in household routines and parental availability.”

2. Introduction

The introduction provides a comprehensive overview of screen time issues in early childhood. However, it would benefit from a clearer description of the questionnaire itself. Specifically, the authors should outline the domains covered by the DSEQ, summarize its previously reported validity and reliability, and briefly explain how the instrument contributes to understanding or mitigating excessive screen exposure. This would better justify the choice of DSEQ and strengthen the study rationale.

**Response:** We have outlined the DSEQ domains, summarized their previously reported validity and reliability, and explained their contribution to the understanding of excessive screen exposure (page 5, lines 100–109).

“The researchers developed a Digital Screen Exposure Questionnaire (DSEQ) for children aged 2–5 years to assess multiple aspects of young children’s screen exposure across five domains, including sociodemographic characteristics, screen time exposure and the home media environment, physical activity, media-related behaviors, and parental media literacy, which showed good validity and reliability and was suitable for use in low- and middle-income countries. This instrument is expected to support researchers in assessing digital screen exposure in early childhood and considering appropriate interventions to reduce screen exposure, particularly in low- and middle-income countries. It may also contribute to the development of age-specific policies for managing digital screen exposure among preschool-aged children.”

3. Methodology
a. Validity assessment: The statistical methods used to evaluate validity are not described in sufficient detail. Content validity typically involves expert evaluation, whereas face validity is usually assessed by non-experts or target respondents. The manuscript should clearly explain: Who was involved in content and face validity assessments, how ratings were performed, the source or framework used for validity evaluation.

**Response:** A new subsection on content and face validity was added (page 8, line 167). Consistent with the original developer study, the validity was evaluated by a multidisciplinary panel of professionals specializing in child development, including pediatrics, child psychology, physical therapy, occupational therapy, speech therapy, and early childhood education (page 8, lines 172–174). The rating method and framework were adopted from a previous study (Kaur, 2021) (page 8, lines 169–171), and detailed explanations of the rating process are provided (page 8, lines 175–179).

The experts were provided with the study objectives and a Likert-type feedback form. Each item was rated on a 4-point scale (1 = “not at all,” 2 = “slightly,” 3 = “moderately,” 4 = “to a great extent”), while the questionnaire length was rated on a 5-point scale (0 = “very short,” 1 = “short,” 2 = “adequate,” 3 = “long,” 4 = “very long”).

The results are presented in Table 3 (pages 18–19).

| Questions | 0 | 1 | 2 | 3 | 4 | Mean |
| --- | --- | --- | --- | --- | --- | --- |
|  | - | Not at all | Minimally | To certain extent | To large extent |  |
| 1. To what extent do you think that the questionnaire will be useful for researchers dealing with children with excessive screen time? |  |  |  | 1 | 11 | 3.9 |
| 2. To what extent do you think that the questionnaire will be useful for caregivers to report the parent perceptions/problems related to screen time? |  |  |  | 5 | 7 | 3.6 |
| 3. To what extent do you think that the questionnaire covers most patterns of usage considered to be associated with screen time? |  |  |  | 5 | 7 | 3.6 |
| 4. To what extent do you think that the questionnaire measures screen time comprehensively? |  |  |  | 8 | 4 | 3.3 |
| 5. To what extent is the language of questionnaire appropriate and understandable? (considering the fact that screen time is common in rural/illiterate and urban/literate population) |  |  | 4 | 8 |  | 2.7 |
| 6. How will you rate the length of the questionnaire? | Very short | Short | Adequate | Long | Very long |  |
|  |  |  | 2 | 9 | 1 | 2.9 |

b. Sample size: The sample size calculation is not adequately described, and no reference is provided. As sample size for validation studies commonly depends on the number of questionnaire items, the rationale and source for the chosen sample size should be explicitly stated and cited.

**Response:** As addressed in response to Reviewer 1, a new subsection describing the sample size rationale with appropriate references has been added to the Methods section (pages 6–7, lines 131–139).

“The minimum required sample size (n = 100) was determined based on the criteria for internal consistency outlined in the COSMIN guidelines [24]. However, a more accurate approach would be to include three to five or up to 10 participants per questionnaire item [25]. A previous study assessed internal consistency using 52 items across domains 2–4 [20]. One item from domain 3 was removed from the online questionnaire (Table 2). Therefore, an internal consistency analysis was conducted using 51 items. Therefore, a minimum sample size of 153 participants was required. The minimum number of participants for test–retest reliability (n = 30) was determined based on a previous study [20].”

c. Test–retest reliability: The choice of a 12-day interval for test–retest reliability is not explained. A brief justification, supported by literature where possible, would improve methodological transparency.

**Response:** We have explained the rationale for the test–retest interval in the Methods section (page 9, lines 192–198). Previous studies have used 10-day and 14-day intervals. In our study, the participants were instructed to complete the retest after 10 days; however, some participants completed it after 11–12 days, which we considered acceptable because it was still within the 10–14-day range. This interval was selected to reduce recall bias while minimizing the likelihood of substantial changes in children’s daily routines.

“The original questionnaire showed test–retest reliability in 30 participants over a 10-day interval [20]. Similarly, another study that developed a questionnaire to assess leisure-time screen-based media use in children aged 6–10 years has reported test–retest reliability in 35 participants over a 2-week interval [28]. In this study, test–retest reliability was assessed with 31 participants over an interval of 10–12 days, which met the minimum sample size requirement and the interval was aligned with previous studies [20,27,28].”

d. Questionnaire distribution: The method of questionnaire distribution to caregivers is not described. Details such as online versus paper-based administration, setting, and supervision should be provided.

**Response:** A new Data Collection subsection has been added to describe the questionnaire’s administration, setting, and supervision (page 7, lines 140–151).

“For convenience sampling, the researcher contacted and met with headmasters or teachers in kindergartens and therapists in pediatric clinics to approach parents who were willing to complete the questionnaire. For snowball sampling, initial participants were asked to recruit or refer other parents who met the study criteria, allowing the sample to expand progressively. The questionnaire was administered online and completed by caregivers in their preferred settings. Caregivers could contact the researcher if clarification was required. As this study involved minors, consent was obtained from their parents or legal guardians. The researcher sent an explanation of the study and an informed consent form in PDF format using WhatsApp. The parents or guardians were asked to read the documents thoroughly before deciding to participate. Those who agreed to participate indicated their consent by clicking on the questionnaire link provided by the researcher and completing the survey.”

e. Additional validity testing: It is unclear whether other forms of validity, such as construct validity or criterion validity, were assessed among participants. If not conducted, this should be acknowledged and justified.

**Response:** We have explained why other forms of validity (e.g., construct or criterion validity) were not assessed (pages 8–9, lines 182–188). Consistent with the original developer’s study, additional validity testing was not conducted because the DSEQ items assessed observable variables rather than latent constructs.

“Furthermore, some validation studies, such as balance-related instruments, have evaluated construct validity, including structural validity (EFA/CFA) and convergent validity [25]. However, consistent with previous research, structural equation modeling (SEM) was not performed because the items in the DSEQ represent observable variables that produce direct outcomes rather than latent constructs [20]. For instance, items in the DSEQ domain assess observable indicators of exposure such as daily screen time duration, screen use frequency, and device availability at home.”

4. Discussion

The discussion would benefit from deeper analytical interpretation of the findings. In particular, the rationale for selecting the 2–5-year age group should be clarified, especially as the questionnaire includes items related to homework, which may not be applicable to younger children. The limitations section should also be expanded to address methodological constraints, generalizability, and areas for future research.

**Response:** The rationale for selecting children aged 2–5 years has been clarified in the Participants subsection of the Methods section (page 6, lines 124–128).

“This age range was selected because the developers of the DSEQ designed the instrument specifically for children aged 2–5 years [20]. In addition, previous studies have reported that the association between excessive screen time and developmental or behavioral problems is stronger in preschool-aged children than in older children or adolescents [14], and that excessive screen time tends to increase with age [7,8].”

Moreover, items related to homework were retained because they originated from the original developer instrument. This study represents the first translation and cultural adaptation of the DSEQ. Retaining these items allowed us to evaluate their relevance in the Indonesian context. In addition, the limitations section has been expanded to address methodological constraints, generalizability, and directions for future research (pages 25 and 26, lines 371–380).

“First, the sample was predominantly drawn from Makassar City, which may limit the generalizability of our findings. Second, the data were collected using convenience and snowball sampling, which may have introduced sampling bias. Third, the screen time data were based on parental reports (questionnaire-based) and may have been subject to recall or reporting bias. However, convenience and snowball sampling and questionnaire-based measures are widely used and considered acceptable in similar contexts, and have been applied in several previous studies on screen time [39,40,41,42]. Moreover, convenience and snowball sampling were employed to facilitate participant access, increase the sample size, and enhance participant trust, particularly because the survey was administered online and link-based scams have become common in Indonesia.”
